# Nondestructive Methods of Pathogen Detection: Importance of Mosquito Integrity in Studies of Disease Transmission and Control

**DOI:** 10.3390/pathogens12060816

**Published:** 2023-06-08

**Authors:** Anne Caroline Alves Meireles, Flávia Geovana Fontineles Rios, Luiz Henrique Maciel Feitoza, Lucas Rosendo da Silva, Genimar Rebouças Julião

**Affiliations:** 1 Laboratory of Entomology, Oswaldo Cruz Foundation, Fiocruz Rondônia, Rua da Beira 7671, Lagoa, Porto Velho 76812-245, RO, Brazil; flavia.rios@fiocruz.br (F.G.F.R.); henrique.feitoza@fiocruz.br (L.H.M.F.); lucas.silva@fiocruz.br (L.R.d.S.); 2 Postgraduate Program in Biodiversity and Health, PhD in Sciences—Fiocruz Rondônia/Oswaldo Cruz Institute, Rua da Beira 7671, Lagoa, Porto Velho 76812-245, RO, Brazil; 3 Postgraduate Program in Experimental Biology—PGBIOEXP, Fiocruz Rondônia—UNIR, BR-364, Km 9.5, Porto Velho 78900-550, RO, Brazil; 4 National Institute of Epidemiology of Western Amazônia—INCT-EpiAmO, Rua da Beira 7671, Lagoa, Porto Velho 76812-245, RO, Brazil

**Keywords:** DENV, *Plasmodium*, vector-borne diseases, entomological surveillance, pathogen detection, routine techniques, artificial membrane assay, feeding substrate, mosquito excreta, integrated taxonomy

## Abstract

Mosquitoes are vectors of many pathogens, including viruses, protozoans, and helminths, spreading these pathogens to humans as well as to wild and domestic animals. As the identification of species and the biological characterization of mosquito vectors are cornerstones for understanding patterns of disease transmission, and the design of control strategies, we conducted a literature review on the current use of noninvasive and nondestructive techniques for pathogen detection in mosquitoes, highlighting the importance of their taxonomic status and systematics, and some gaps in the knowledge of their vectorial capacity. Here, we summarized the alternative techniques for pathogen detection in mosquitoes based on both laboratory and field studies. Parasite infection and dissemination by mosquitoes can also be obtained via analyses of saliva- and excreta-based techniques or of the whole mosquito body, using a near-infrared spectrometry (NIRS) approach. Further research should be encouraged to seek strategies for detecting target pathogens while preserving mosquito morphology, especially in biodiversity hotspot regions, thus enabling the discovery of cryptic or new species, and the determination of more accurate taxonomic, parasitological, and epidemiological patterns.

## 1. Introduction

Mosquitoes, together with hard ticks, are the main arthropods responsible for the transmission of emerging vector-borne diseases (VBDs), chiefly caused by RNA viruses and Rickettsiaceae bacteria, respectively [1,2]. VBDs are also disseminated by sand flies, kissing bugs, fleas, flies, mites, lice, and snails, and account for around 17% of the estimated global burden of infectious diseases. Around 80% of the world’s population are at risk of contracting one or more vector-borne diseases, while more than 50% are at risk from two or more VBDs [3]. The name “mosquito” was established worldwide for dipteran insects of the family Culicidae. It is derived from Spanish or Portuguese, most likely on the American continent, and means “little fly” [4].

In addition to a wide variety of viruses, mosquitoes have been incriminated as vectors of malaria protozoans (*Plasmodium* spp.) and filarial worms (*Brugia* spp. and *Wuchereria bancrofti*). Approximately 300 viruses have been recorded in mosquitoes, of which about 100 can be transmitted to humans [5,6]. Many mosquito species of the Culicidae family significantly impact human health, such as members of the genus *Aedes*, which are the vectors of dengue (DENV), chikungunya (CHIKV), and Zika (ZIKV) viruses, and *Anopheles*, the main malaria vectors [5,6].

Due to the health emergencies that some vector-borne diseases have caused, medical and veterinary entomology has become an increasingly important area of knowledge [7]. Despite its relevance worldwide, it faces many bottlenecks due to the disparity of time and resources invested in training professionals in the field, and insufficient and/or intermittent funding, resulting in a shortage of taxonomists and medical entomologists [8,9]. These are highly skilled professionals who require intensive training to be able to perform several methods of vector identification, monitoring, and research.

Entomological and parasitological surveillance is mainly based on concomitant field and laboratory investigations on vector density, biting activity, host preference, vector competence, and vectorial capacity [7,10]. In turn, the detection of pathogens in the vectors also requires other technical skills, and usually employs destructive sampling procedures with whole mosquitoes or their body parts, such as the head, thorax, salivary glands, and guts, as a basis for inferring pathogen infection, dissemination, and transmission [7,10].

Some alternative surveillance programs for mosquitoes have been proposed, including citizen science [11,12,13] and the metabarcoding of bulk samples [14,15,16], for assessing and monitoring vector density at a given location, time, or season. Mosquito metabarcoding faces challenges regarding the cost per sample, as well as an absence of sequences for known taxa, or sequences recorded for unknown taxa, in addition to the standardization of datasets [17,18]. In spite of these shortcomings, multiple citizen science initiatives have been integrated, resulting in the creation of the Global Mosquito Observations Dashboard (GMOD) platform, to provide rapid and integrative information on mosquito populations and/or distribution [13]. VectorBase (https://vectorbase.org/; accessed on 18 March 2023), another integrative platform, combines vector arthropod and pathogen databases, including their genomes, genetic variation, and functional genomic approaches. Several tools, such as interactive graphs and maps, allow for data visualization, search, and analysis [19].

Thus, even with the advances obtained through integrated taxonomy using molecular tools and markers, such as the COI gene (cytochrome c oxidase subunit I) [20,21,22,23], classical taxonomy based on morphology is still considered the golden standard for vector incrimination and control, which ultimately leads to the interruption of disease transmission. In this sense, the specimen value is enhanced, mainly if deposited in scientific entomological collections, where its additional records can be assessed, supporting research on Culicidae diversity or providing information on introduced and invasive species [24,25]. Here, we performed a brief literature review to explore the importance of mosquito taxonomy and systematics, some examples of viruses, bacteria, protozoa, and helminths transmitted by these insects, and noninvasive and nondestructive techniques for pathogen surveillance and identification.

The purpose of our study was to compile alternative strategies for detecting pathogens while preserving mosquito morphology, which can allow for further research on cryptic or new species, mainly in biodiversity hotspots, such as the Amazon biome [20,26,27,28,29,30], where there are scant reports on these insects [31,32]. These nondestructive techniques may now, or in the future, contribute to maintaining the basic requirements of taxonomic and parasitological studies, both to correctly identify vector species and to gather data on the natural rate of infection or the transmission of target pathogens.

## 2. Why Do Mosquito Systematics Matter?

Taxonomy and systematics are scientific fields that aim at the classification and organization of living organisms. Taxonomy classifies organisms based on different morphological and physiological characteristics to create a nomenclature that allows for their identification [33]. Systematics establish the phylogenetic relationships between extant organisms and their ancestors [34]. The construction of phylogenetic trees illustrates the evolutionary history of living beings and the characteristics shared between species [35]. The term “species” corresponds to biological entities based on a taxonomic reference system, with scientific names used for their identification [36,37,38].

Medical and veterinary entomology plays a fundamental role in the identification of arthropods, as (i) the correct identification of species that transmit pathogens facilitates the adequate understanding of patterns of transmission and the distribution of diseases, and (ii) they provide baseline information for programs of entomological and epidemiological control [39]. If incorrect entomological identification occurs, it may result in high costs and consequences to public health [40]. A debate has been ongoing for several decades regarding the need for and the call to fill the gap in insect taxonomy and systematics [41], especially with insect vectors [42].

### 2.1. Mosquito Systematics and Taxonomy

The quality of taxonomic data is an issue that has recently been raised. Data of unknown quality are frequently used, resulting in a cascade of inaccurate information. It has therefore been recommended that uncertainties encountered in taxonomic identification are documented and reported [43,44,45].

In addition to morphological identification, molecular methodologies, such as PCR coupled with DNA sequencing, proteomics, and isoenzyme analysis, have been used to identify mosquito species [46,47]. Molecular tools and analyses are especially important to distinguish morphologically similar species. The banding pattern of chromosomes, isoenzyme profiles, DNA probes, the analysis of DNA restriction fragments, cytogenetic physical methods, the analysis of nucleotide sequences of target genes, and barcoding and metabarcoding have been employed as routine molecular methods [6,15,17].

More recently, barcoding and metabarcoding have been demonstrated as successful tools in mosquito taxonomy tasks. In Brazil, the D2 extension segment of the 28S ribosomal DNA (D2 rDNA) was found to be a reliable marker for species identification within mosquito assemblages based on mock genomic DNA pools [14]. Immature and adult Culicidae were classified into species using this marker. For adult mosquitoes, the D2 rDNA approach was comparable to morphological diagnosis. However, several immature forms of specimens of the genus *Mansonia* could not be classified into species [15]. Researchers in Canada, using DNA metabarcoding based on the COI gene, were able to identify and also estimate the number of captured mosquitoes. While 33 species of mosquitoes were classified using the metabarcoding approach, only 24 species were identified using morphological keys [16].

However, morphological identification remains the preferred method for both scientific research and surveillance since it can easily be implemented in the field and requires little technical equipment, in addition to being less expensive when there is a large number of mosquitoes to be identified [48].

Identification based on morphological characteristics has three main limitations: (I) the dependence on experienced entomologists, (II) reliance on the intactness of the specimen, since loss of body parts can make identification impossible, and (III) cryptic and morphologically similar species, whose identification is based on more than one developmental stage, which is often not easily obtained [48].

### 2.2. Classical Taxonomy x Integrated Approaches

Classical taxonomy is based on the use of morphological characteristics such as size, color, and specific body structures to classify organisms [49]. This taxonomic method has been widely used in the past and is still essential in species identification.

Different authors have used the term “integrative taxonomy” independently [49,50]. Although divergent opinions exist, this method works by analyzing several insect characteristics, including target sequences from their nuclear and/or mitochondrial DNA [51,52].

Due to the speciation process, some morphological alterations go unnoticed during identification [53], and the use of classical taxonomy in combination with other integrative methodologies, such as cytospecies characterization (chromosomal analysis), isoenzyme profiles (biochemical analysis), gas chromatography (cuticular hydrocarbons analysis), scanning microscopy, morphometry, and molecular biology, has been used to assist in the differentiation of cryptic species [5,10,54]. Molecular tools comprise a variety of techniques, such as DNA hybridization assays and polymerase chain reaction (targeted PCR and RAPD) coupled, or not, with DNA sequencing. With these complementary techniques, it is possible not only to identify and differentiate species but also to study their genetic variability and infer their phylogeny [5,10,54].

The use of Integrative tools, combined with the morphological characterization of mosquitoes from all developmental stages, could represent a breakthrough in the comprehension of phylogenetic relationships between similar species, as observed for infrageneric groups of Aedini mosquitoes [55]. In addition to assisting in the identification of species complexes, as well as understanding the evolutionary processes in the Culicidae taxa, molecular tools can also be applied to studies of speciation events, resistance to insecticides, pathogen identification, and genetic engineering for vector control purposes [10,56].

### 2.3. Culicidae as a Biodiversity Component

The family Culicidae currently has more than 3,500 species worldwide, classified into two subfamilies (Anophelinae and Culicinae), that play key roles in terrestrial, freshwater, and marine ecoregions [6,57,58]. These insects occupy all available niches due to their immense morphological variation and adaptability, characterizing them as the most successful beings on the planet [59], and are remarkably diverse in tropical forests [60,61].

There has been a growing interest in these insects due to their ability to transmit arboviruses and protozoa responsible for causing diseases to both humans and animals [62].

## 3. Pathogens Transmitted by Mosquitoes

Recent findings expand the list of mosquito species associated with main VBDs, but the gap in knowledge about their vectorial capacity in the field persists [3,63] (Table 1).

### Life Cycle of Parasites and Viruses in the Mosquito

Many pathogens, such as malaria parasites and some viruses, spread to humans and other animals through the “mosquito bridge”. After a blood meal on an infected vertebrate host, the pathogen must pass through several physical and immunological barriers in the mosquito midgut, hemocoel, and salivary glands to reach the proboscis and be expelled with saliva, sustaining the pathogen transmission cycle [83,84].

For a virus, the first tissue it contacts after blood ingestion by a mosquito is in the midgut. The virus then passes through the basal lamina of the midgut epithelium and migrates to its cells, where it disseminates throughout the insect tissues. Depending on the type of virus, it can reach fatty, nervous, and muscle tissues, as well as hemocytes. Finally, the virus reaches the salivary glands (the last barrier before transmission of the pathogen), where it replicates. After replication in the apical cavities of the acinar cells, virus particles are then expelled with saliva upon mosquito feeding on another vertebrate host [83,84].

The extrinsic incubation period refers to the time required for the virus to reach the salivary glands after it is ingested in a blood meal. It lasts 8 to 12 days in the case of DENV, a time that varies for other virus species [84,85].

Concerning protozoan pathogens, such as *Plasmodium* spp., they need their invertebrate host for development, with reproduction occurring during the sporogonic cycle (10 to 18 days) in the mosquito. This cycle begins when a mosquito ingests gametocytes in a blood meal. The gametocytes mature into female and male gametes, giving rise to the fertilization process (sexual reproduction), forming a zygote, which further differentiates into an ookinete. The latter crosses the peritrophic matrix, traverses the midgut wall, and develops into oocysts, which, upon maturation, release sporozoites. At this stage, the parasite migrates to the hemocoel and infects the salivary glands. Then, it is eventually released during hematophagy, thereby restarting the parasite life cycle in the new vertebrate host [83,86].

It Is worth mentioning that the microscopical observation of a pathogen or the detection of its DNA/RNA within a mosquito sample does not necessarily mean that the carrying species is the vector transmitting the pathogen. Evidence for vector competence is still required, since not all mosquitoes with an ingested pathogen are suitable for pathogen replication, dissemination, and transmission, or survive long enough for the full developmental cycle of the pathogen to occur [7].

## 4. Nondestructive Approaches to Pathogen Detection in Mosquitoes

In order to control and reduce the impacts of VBDs, several indexes have been proposed for monitoring disease transmission and its geographical and temporal extent. Research and surveillance programs have yielded and included some metrics based on environmental, entomological, or epidemiological variables. The pathogen prevalence and incidence in humans and animals are the standard metrics employed in most surveillance programs. However, some more robust approaches have also been performed in mosquito sampling and analysis of its infective status [10].

Current gold-standard techniques for pathogen detection and vector incrimination in mosquitoes result in damage to the insect body. For instance, the analysis of malaria sporozoites in the salivary gland is performed via microscopic dissection or PCR, both resulting in the destruction of insect body and precluding its identification through many of the standard taxonomical procedures.

Nondestructive techniques for pathogen detection are summarized below, based on the recovery and detection of pathogen genetic material, in mosquitoes collected from the field or in laboratory assays. In these alternative techniques, basic aspects of mosquito biology are considered. In nature, adult forms of Culicidae, of both sexes, first feed on sugary solutions (plant nectar or honeydew) for obtaining energy for sexual maturation, flight dispersion, mating, and finally, host finding. In the latter, only female insects feed on vertebrate blood for egg development and maturation, and offspring establishment [5,6].

**Figure 1:**
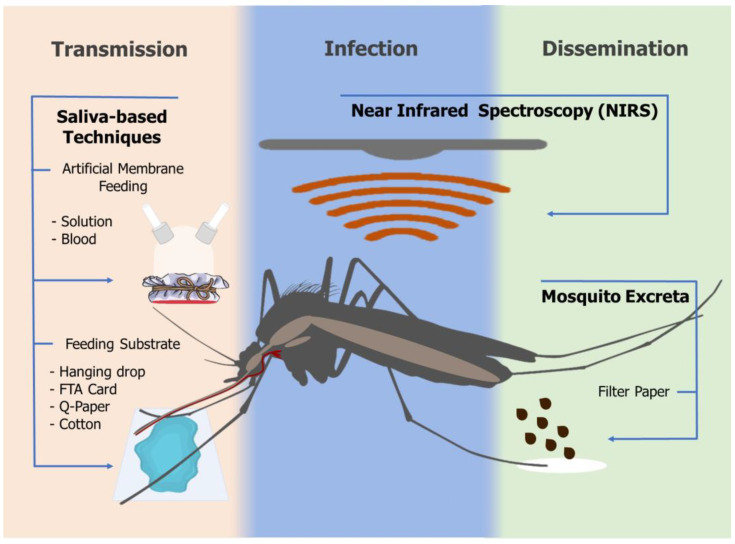
Schematic representation of nondestructive techniques for pathogen detection or recovery.

Three main nondestructive techniques for pathogen detection, taking into account mosquito biology, are described: (1) saliva analysis of an infected mosquito during its blood feeding, when it is already capable of transmitting a pathogen; (2) mosquitoes refractory to infection, but expelling parasites in their feces; (3) whole-body analysis of mosquitoes via near-infrared spectrometry, for evaluation of pathogen infection and dissemination (Figure 1).

### 4.1. Saliva-Based Technique—Feeding Substrate

#### 4.1.1. FTA Cards

Flinders Technology Associates (FTA) cards developed by Whatman plc (GE HealthCare Technologies Inc., Chicago, IL, USA) are widely used for sample preservation and purification, enabling the analysis of genetic material from various types of biological sample, such as blood and other fluids, tissues and cells. This card is made up of filter paper containing chemicals to lyse cells, denature proteins, and prevent bacteria growth. The nucleic acids are immobilized and preserved on the card matrix and can be extracted using various purification methods [87,88,89].

Aside from stabilizing biological samples from various sources, the card can be kept at room temperature for a few weeks without requiring any special storage conditions. This sampling alternative has been shown to be promising for virological surveillance, due to its low-cost, long-term storage in the field, and stabilization of viral RNA for several days and at different temperatures, which are all necessary for maintaining the integrity and stability of samples of interest [88,89,90].

Several research groups have used this card with sugar as bait, to detect arboviruses in the mosquito saliva released during sugar-based feeding. FTA cards inactivate any expectorated virus during mosquito feeding and can be used with sugar bait, which is usually made of a diluted honey solution [66,90,91,92,93,94,95]. In addition to being simple and effective, FTA cards can also be applied to field traps, minimizing the cost of field collection and storage, and the processing of the large number of mosquitoes that is necessary in such surveillance [90,91,96,97,98].

Several arboviruses have already been detected in field mosquitoes using this approach. Ross River (RRV) and Barmah alphaviruses were detected in two locations in Australia [90], and CHIKV in French Guiana [98]. Natural DENV infection in *Ae. aegypti* saliva was recently demonstrated in the Brazilian Amazon in infected female mosquitoes fed on honey-soaked FTA cards [66]. ZIKV released in the saliva by *Cx. quinquefasciatus* and *Ae. aegypti* was also detected, even with low viral loads [94]. The FTA card strategy has been successfully implemented for screening other pathogen taxa, e.g., malaria sporozoites expelled by female *Anopheles* infected with *Plasmodium berghei* during feeding on FTA cards soaked with glucose solution [99].

In the laboratory, standard filter paper soaked with honey solution (50%) has also been used for detecting pathogens in mosquito saliva, allowing for the molecular detection of RNA from several arboviruses. After oral infection of *Ae. aegypti* and *Ae. albopictus* with CHIKV, viral RNA was detected after up to 7 days on the filter paper [100]. WNV was also efficiently recovered on filter paper strips after experimental infection assays of *Culex tarsalis* [101]. Similarly, nucleic acids of protozoan samples can be obtained from saliva ejected onto standard filter paper. The saliva deposited by infected *Anopheles stephensi* on honey-soaked filter paper contained *Plasmodium falciparum* DNA [102].

A refined honey-soaked filter paper method was developed by including red food dye, and was used to detect RRV and WNV expectorated in the saliva of *Aedes vigilax* and *Cx. annulirostris*, respectively [103]. Food dye has also been used to record whether mosquitoes have been fed the sugary solution on paper or cotton substrates, by analyzing their crop and excreta [66,94,102]. This dyeing procedure also allowed for the estimation of ingested solution volume, both with the naked eye and via spectral absorbance [104].

#### 4.1.2. Q-Paper

The cationic Q-paper (Q-paper) is a substrate with a cationic surface composed of cellulose containing quaternary ammonium groups, developed primarily to absorb poisonous compounds from wastewater [105]. It can be used to collect, preserve, and store nucleic acids from different biological samples containing, for instance, viruses, and nucleic acid then binds electrostatically to this substrate [106]. The amount of genetic material recovered from the Q-paper may vary depending on its binding capacity, and depending on the paper composition [107].

The Q-paper method has been used to assess the ability of some mosquito species to transmit arboviruses. The potential for the transmission of MAYV by *Ae. aegypti* and *Ae. albopictus* was demonstrated through their expectorated saliva on Q-paper treated with a 20% sucrose solution [108]. DENV-2, CHIKV, and ZIKV have also been detected on Q-paper with a honey solution and blue dye exposed to experimentally infected *Ae. aegypti* [107,109].

#### 4.1.3. Cotton Substrates

Saliva-pathogen ejection during mosquito feeding has also been explored using a simpler and less expensive cotton-wool wick soaked in sugary solution. It was used to collect saliva from *Cx. tarsalis* previously infected with three strains of WNV. The procedure was compared to the methods of using filter paper strips and capillary tubes, the latter of which is a standard technique in which a mosquito proboscis is inserted into a glass or plastic microcapillary, and is also known as the forced salivation technique. There was no statistical difference in virus recovery between cotton and the capillary tube, while more virus RNA was recovered from the cotton wick than the filter paper, demonstrating that the cotton wick can also be an efficient alternative for verifying virus transmission [101,110].

In addition to viruses, cotton wicks were also used to detect *Plasmodium falciparum* in infected mosquito saliva from five *Anopheles* species (*An. gambiae*, *An. coluzzii*, *An. arabiensis*, *An. stephensi*, and hybrids of *An. gambiae*/*An. coluzzi*). The infected mosquitoes were fed on small pieces of cotton soaked in a sugar solution. Parasite DNA from sporozoites expelled with the saliva was detected via qPCR. Sensitivity was high but less than 100%, which could be because infectious females either expelled undetectable levels of sporozoites on the cotton, especially in the initial days after infection, or had not fed on the sugar solution, a fact already observed in other methods [111].

#### 4.1.4. Hanging Drop Method

In general, this method consists of feeding one or several droplets of about 0.025 mL of blood solution to mosquitoes experimentally infected with arbovirus. The blood solution is usually prepared with defibrinated blood plus sucrose, or erythrocyte and serum, or erythrocyte plus fetal calf serum (FCS) and sucrose suspensions. After mosquito feeding and/or probing, the drops are mixed with phosphate-buffered saline and FCS, and assayed for virus detection, in order to evaluate mosquito transmission ability [112,113,114,115].

This technique, although simple and inexpensive, has been proven to be less efficient in comparative studies due to the lack of host stimuli for probing and feeding, and hence, underestimates the potential for virus transmission by a mosquito species [114,115,116]. Nevertheless, this method has been proven successful in other studies involving vector competence. In one study, ten mosquito species were infected with WNV, and, using the abovementioned technique, the researchers were able to observe differences in transmission rates between the mosquito species [117]. WNV infection was also detected in 42% of *Cx. annulirostris* mosquitoes using the hanging drop method in the experimental assays [103].

Future studies could improve this technique with the inclusion of chemical compounds in the blood suspension, which stimulate mosquito probing, feeding, and full engorgement.

### 4.2. Saliva-Based Technique—Artificial Membrane Feeding (AMF)

Mosquito rearing in laboratory conditions has several applications in entomological and epidemiological research on their basic biology, vector competence, control strategies, and resistance to insecticides. However, a challenge for mosquito colony maintenance is female egg production, which depends on blood for insect development [118, and references herein]. An artificial membrane feeding (AMF) system, as an alternative to feeding on vertebrate hosts, has been developed to provide blood meals for mosquitoes reared in the laboratory for colony maintenance and experimental infections. An AMF system consists of a mosquito cage connected to a container (of glass, plastic, or paper materials) with human and/or animal blood and covered with a natural or artificial membrane. Several mosquito-feeding devices have been developed and modified according to different purposes. The efficiency rate of AMF depends on the components adopted, often related to the cost and availability of materials and blood from a given host, as well as the enrichment with phagostimulants [118].

#### 4.2.1. AMF—Blood-Feeding

The blood-feeding salivation test is performed by exposing infected mosquitoes to uninfected blood through AMF. Subsequently, the blood sample containing mosquito saliva is analyzed for detecting specific pathogens. Recently, this approach was used to detect DNA of *Plasmodium vivax* from the saliva of experimentally infected *Anopheles* mosquitoes. Sporozoite DNA was successfully traceable to all six species: *An. triannulatus*, *An. nuneztovari*, *An. benarrochi*, *An. evansae*, *An. aquasalis*, and *An. darlingi*; the highest infection rate was recorded for the latter species, the primary mosquito vector in the Amazon region [119]. On the other hand, viral RNA was rarely detected in AMF blood samples used to feed *Ae. aegypti*, previously infected with ZIKV and CHIKV, although both viruses were detected in saliva using a capillary tube method [120].

#### 4.2.2. AMF—Non-Blood-Feeding

An alternative allure to blood for collecting saliva from mosquitoes was used that consisted of a solution of phosphate-buffered saline (PBS) and adenosine triphosphate (ATP). It was successfully used to collect saliva from *Aedes* mosquitoes infected with DENV-2. This solution in AMF apparatus allowed for the collection of saliva from multiple mosquitoes at the same time, in addition to the detection of viruses at low concentrations as early as the 7th day after infection [121].

### 4.3. Mosquito Excreta

An alternative nondestructive approach, recently used in molecular parasite surveillance of pathogens is the analysis of mosquito excreta/feces, which sample volumes are usually larger than those of saliva [103]. This method makes it possible to detect and recover pathogens or their genetic material, both from competent and non-competent vector species. For instance, non-developed parasites (*Brugia malayi*) were found in the feces of non-competent mosquitoes, with implications in the design and interpretation of molecular diagnostic of pathogens in arthropod vectors [122]. Likewise, with viruses, experimental infection studies showed, in addition to higher sensitivity, a greater proportion of viral RNA detection in excreta samples than in saliva samples [103,123].

A method using a superhydrophobic cone (SHC) made of A4 printer paper, and coated with a hydrophobic water repellent (NeverWet^®^, NeverWet, LLC), has been developed to collect mosquito excreta/feces (EF) for further analysis of pathogen DNA/RNA. The SHC is inserted into a paper cup, so the feces are deposited on an FTA card or in a 1.5 mL tube at the bottom of the cup. The feces are then recovered via direct washing or wet swabbing. The mosquitoes were experimentally infected with *P. falciparum*, *Brugia malayi*, and *Trypanosoma brucei brucei*. The collection methods enabled DNA detection for all three parasite species in the EF [124]. *B. malayi* was detected in the vector (*Ae. aegypti*), as well as in the EF of a non-vector mosquito (*Cx. pipiens* s.l.), when exposed to the parasite [122]. It is worth noting that the SHC combined with a microcentrifuge tube was the method that gave the highest number of positive samples.

Three parasites, *W. bancrofti*, *P. falciparum*, and *Mansonella perstans*, were detected in field mosquito excreta using the combined SHC+tube method [125]. This promising method has shown great potential for the surveillance of other circulating pathogens. Recent findings of xenosurveillance demonstrated the presence of *Loa loa* filaria (the causative agent of loiasis) in three EF samples obtained from wild mosquitoes, in addition to *W. bancrofti*, *M. perstans*, and *P. falciparum* [126].

The analysis of mosquito excreta has also been widely used for arbovirus detection and surveillance. After 15 days, viral RNA was successfully detected in excreta deposited on filter paper by female *Ae. aegypti* infected with DENV [123]. WNV was detected in the excreta of infected *Cx. annulirostris*, deposited on two substrates: an FTA card and a polycarbonate disk. Viral RNA was recovered for up to 2 weeks, even under high temperatures and humidity [127]. Continuous positivity from the 2nd day to the 15th after infectious feeding was obtained in the excreta of *Cx. annulirostris* and *Ae. vigilax*. Specimens of these species were infected with WNV and RRV, respectively. Mosquito excreta was then deposited on a parafilm M disk and removed with moistened cotton swabs [103]. Using this method, WNV was also detected in the excreta of naturally infected field mosquitoes. Samples were obtained from captured mosquitoes on filter paper inserted in a BG Sentinel trap. This strategy of pathogen xenomonitoring is considered simple, effective, and economical, enabling inferences about the circulation of arboviruses, the density of insect vectors, and potential vertebrate hosts [128].

This alternative strategy has some limitations, however. DNA integrity might be lost due to the mosquito digestion process and due to environmental exposure. Additionally, exogenous DNA contamination is a possibility, especially with field mosquitoes or the use of contaminated materials and devices. In the case of a parasite, its life stage cannot be determined. Application in the field also requires the initial grouping of mosquitoes by species to allow for better inferences regarding vector, host, and pathogen relationships [102,128].

### 4.4. Near-Infrared Spectroscopy (NIRS)

NIRS is a high-throughput automated analytical technique that measures tissue light absorption at various wavelengths in the spectral region between 750 and 2500 nanometers (nm). The emitted waves interact with biological samples and are absorbed by bonds of specific chemical molecules (e.g., CH, NH, SH, or OH). The absorption is measured using a spectrometer from which, after data analysis, a sample spectrum can be obtained [129,130]. Near-infrared spectrometry is currently applied in studies investigating the age structure of mosquito populations, as a complementary method [131,132], as well as in comparisons between mosquitoes exposed to insecticides [133] and different diets [134], in addition to estimating the parity status of females [135].

The NIRS technique has recently been used to detect pathogens in experimentally infected mosquitoes. The results with DENV, ZIKV, and CHIKV have revealed satisfactory accuracy generally above 90% [136,137,138]. The inclusion of more robust computational analysis has improved the accuracy of the technique. The use of computational genetic algorithms (GA) and linear discriminant analysis (LDA) improved to 100% sensitivity and specificity in DENV detection in *Ae. aegypti* with recent infection [139].

A malaria parasite (*P. falciparum*) experimentally infecting *An. gambiae* was detected with an accuracy of 88% for oocysts and 95% for sporozoites [140], while an efficiency lower than 75% was found in laboratory-reared *An. coluzzii* infected with the parasite. NIRS models based on experimental assays were not able to predict the natural infection of field mosquitoes [141]. Variations in the age of field mosquitoes may be the primary explanation for the inability to detect infection. Predicting age parameters for field mosquitoes is a previously reported challenge [142]. The findings obtained by Da et al. [141] corroborate another previous study where *An. stephensi* mosquitoes were infected with *P. berghei* [143].

NIRS seems very promising and has several advantages, such as a non-invasive procedure, minimal requirements for prior sample preparation, fast results, no necessity for reagents or multifunctional devices that require long-term experience and training, and being less expensive than the currently used methods in pathogen investigation [130]. However, NIRS still needs adaptation and improvements, both in chemometric approaches and computational analysis, to allow for its application in entomological and pathogen research.

## 5. Scientific Horizons Expanded: Museums, Biorepositories, and Nondestructive DNA Extraction

Voucher specimens are crucial for repeatability and extension in epidemiological research since they permanently preserve records and samples from pathogens, hosts, and vectors (as well their cells and tissues), which can be further reached and reanalyzed [144,145]. Undeniably, museums and biorepositories are valuable sources of sample materials and their associated databases, with direct application in ecological and epidemiological studies, shedding light on patterns in the transmission of infectious diseases, emerging or novel zoonotic pathogens, and their reservoir hosts and vectors [146,147].

However, molecular analysis of pinned and dried mosquitoes from museums and collections are not an easy task. Maintaining their taxonomic identity requires the integrity of their external morphology, arranged in detail with scales and bristles, while molecular methodologies require DNA with adequate quantity and quality [148,149].

Electronic vouchering and genome sequencing pipelines have been tested for mosquitoes and triatomines [150]. The same DNA extraction protocol was employed for these two vector groups, which was nondestructive for kissing bugs, which are usually larger than mosquitoes and without scales. For mosquitoes, damage was inevitable and specimen selection criteria were established. Thus, studies that have museums and collections as a source of material must consider the balance between the need to apply molecular tools and the preservation of the specimen integrity [150]. Alternatively, the destructive extraction of genomic DNA from mosquito eggshells and larval and pupal exuvium, which were reared to adulthood in a laboratory, helped to identify Culicidae species via PCR profiling [151].

“Mild-Vectolysis” [148] was the first nondestructive DNA extraction protocol established for mosquitoes and sand flies, allowing for DNA barcoding while vouchering the specimens. To obtain sufficient mosquito DNA, some steps in the protocol were adjusted, such as the concentration of lysis buffer, incubation time, post-lysis freezing stage, and avoidance of ethanol, before starting the lysing step. Using the adjusted method, DNA recovered from all mosquito samples was enough for amplifying a fragment from their COI gene. There was no damage to the mosquito’s exoskeleton and appendages, except some loss of scales and tegument color [148]. To date, no attempts have been registered to optimize this promising methodology, which may further advance taxonomical and parasitological studies of mosquito vectors.

## 6. Conclusions

This review highlights the need of alternative strategies for the surveillance of vector-borne pathogens that preserve insect integrity, which is critical for the discovery of vectorial capacity in cryptic or new insect species, especially in biodiversity hotspot areas. Alternative methods have produced contrasting results, and the need for the standardization of techniques, as well as the materials used within a method, is apparent. Few studies have been performed on the use of these alternative methods on a larger set of vector species and their application in the field while taking into account broader temporal and geographic scales. Further research should focus on method comparisons regarding their efficiency, sensibility, reliability, and reproducibility in parasite detection, prioritizing feasible and less expensive nondestructive techniques.

## Figures and Tables

**Table 1:** Mosquito species naturally carrying pathogens and their primary arthropod vectors.

Group	Pathogen Species	Main Vector Species ^1^	Vector Mosquito Species ^2^	Country or Continent ^3^	References ^4^
Virus	DENV	*Ae. aegypti*, *Ae. albopictus*	*Cx. quinquefasciatus*, *Cx. bidens*, *Cx. interfor*, *Psorophora* spp., *Ps. varipes*, *Ps. albigenu*, *Sa. chloropterus*	Brazil	[64,65]
	ZIKV	*Ae. aegypti*, *Ae. albopictus*	*Cx. quinquefasciatus*, *Hg. leucocelaenus*	Brazil	[64,66,67]
	CHIKV	*Ae. aegypti*, *Ae. albopictus*	*Cx. quinquefasciatus*, *Ps. ferox*, *Ps. albigenu*	Brazil	[66]
	YFV	*Haemagogus* spp., *Sabethes* spp., *Aedes* spp.	*Ae. albopictus*, *Ae. aureolineatus*, *Sa. identicus*, *Sh. fluviatilis*	Colombia	[67,68]
	MAYV	*Haemagogus* spp.	*Ae. aegypti*, *Ae. albopictus*, *Cx. quinquefasciatus*	Brazil	[66,69]
	OROV	*Culicoides paraensis* (biting midge)	*Cx. quinquefasciatus*, *Cq. venezuelensis*, *Oc. serratus*, *Ps. cingulata*, *Hg. tropicalis*	Brazil	[70,71,72]
	VEEV	*Cx. (Melanoconion)* spp.	*Ae. scapularis*, *Ae. aegypti*, *Ae. albopictus*, *Culex* spp., *De. atlanticus*, *Ma. titillans*, *Ps. confinnis* *Haemagogus* spp., *Sabethes* spp., *Deinocerites* spp., *Anopheles* spp.	Brazil, Colombia	[70,73]
	SLEV	*Cx. quinquefasciatus*, *Cx. pipiens* s.l., *Cx. nigripalpus*, *Cx. tarsalis*	*Culex* spp., *Ma. Titillans*	Colombia	[73,74]
	WNV	*Cx. pipiens* s.l., *Cx. univittatus*	*Culex* spp., *Cx. perexiguus*, *An. Crucians*, *An. Quadrimaculatus*, *Cq. Perturbans*, *Cx. coronator*, *Cx. erraticus*, *Cx. nigripalpus*, *Cx. quinquefasciatus*, *Ma. Titillans*, *Ae. sollicitans*, *Ps. Columbiae*, *Ur. Lowii*	Colombia, Spain, Algeria, USA	[73,75,76,77]
	JEV	*Culex tritaeniorhynchus*, *Culex annulirostris*	*Aedes* spp., *Anopheles* spp., *Ar. Subalbatus*, *Cq. Ochracea*, *Culex* spp., *Mansonia* spp.	Australia/Oceania, Europe, Asia	[78]
	USUV	*Cx. pipiens* complex	*Ae. albopictus*, *Cx. neavei*, *Cx. quinquefasciatus*, *Ae. japonicus*, *Ae. vexans*, *An. Maculipennis*, *An. Plumbeus*, *Cq. Richiardii*, *Cs. annulate*, *Ochlerotatus* spp.	Europe	[79]
Bacteria	*Rickettsia felis*	*Ctenocephalides felis* (flea)	*An. Sinensis*, *Cx. pipiens* s.l., *Ae. albopictus*, *Ar. Subalbatus*	China	[80]
	*Borrelia burgdorferi* complex	*Ixodes* spp. (tick)	*Aedes* spp., *Culiseta* spp., *Culex* spp., *Ochlerotatus* spp.	Germany	[81]

DENV: dengue virus, ZIKV: Zika virus, CHIKV: chikungunya virus, YFV: yellow fever virus, MAYV: Mayaro virus, OROV: Oropouche virus, VEEV: Venezuelan equine encephalitis virus, SLEV: Saint Louis encephalitis virus, WNV: West Nile virus, JEV: Japanese encephalitis virus, USUV: Usutu virus. ^1^ Main arthropod vector detailed by Foster and Walker [6] and Marcondes [70]. Abbreviation of mosquito genera following Reinert [82]. ^2^ Mosquito species found naturally carrying pathogens, but with no evidence of their transmission ability in nature and/or laboratory. ^3^ Country or Continent: geographic region where the mosquito species carrying a pathogen have been recorded. ^4^ Report of field mosquito carriers but without evidence of transmission competence.

## Data Availability

Not applicable.
